# Optimistic biases in observational learning of value

**DOI:** 10.1016/j.cognition.2011.02.004

**Published:** 2011-06

**Authors:** A. Nicolle, M. Symmonds, R.J. Dolan

**Affiliations:** Wellcome Trust Centre for Neuroimaging at UCL, 12 Queen Square, London WC1N 3BG, UK

**Keywords:** Learning, Social cognition, Decision making, Value, Reward

## Abstract

Action-outcome contingencies can be learnt either by active trial-and-error, or vicariously, by observing the outcomes of actions performed by others. The extant literature is ambiguous as to which of these modes of learning is more effective, as controlled comparisons of operant and observational learning are rare. Here, we contrasted human operant and observational value learning, assessing implicit and explicit measures of learning from positive and negative reinforcement. Compared to direct operant learning, we show observational learning is associated with an optimistic over-valuation of low-value options, a pattern apparent both in participants’ choice preferences and their explicit post-hoc estimates of value. Learning of higher value options showed no such bias. We suggest that such a bias can be explained as a tendency for optimistic underestimation of the chance of experiencing negative events, an optimism repressed when information is gathered through direct operant learning.

## 1 Introduction

Many instances of everyday learning rely upon trial-and-error. Here, a decision-maker samples between alternative actions and risks unfavorable outcomes in the early stages of learning, when action-outcome contingencies are unknown. Learning can also occur through observing the successes and failures of others, enabling us to acquire knowledge vicariously. Indeed, the benefits of observational learning are ubiquitous in nature. For example, a hungry animal can avoid the energy costs incurred in active sampling of optimal feeding locations by observing actions and outcomes of conspecifics. A proliferation of customer review websites epitomizes the utility of learning through the positive and negative experiences of others so obviating our own need for expensive decisions. In this way, observational learning is recognized as supporting “locally adaptive behaviors without incurring the costs associated with individual learning” (Boyd & Richerson, 1988, p. 30).

Surprisingly, the efficacy of observational learning has been rarely studied in the context of human value learning. Empirical evidence in animals attests to the fact that rewarded behavior is promoted, and punished behavior diminished, in passive observers (e.g. Bandura, 1971; Dawson & Foss, 1965; Heyes & Dawson, 1990; Mineka & Cook, 1988; Weigl & Hanson, 1980). For example, budgerigars show imitation of rewarded behaviors but a diminution of such behavior if the observed consequences are not salient, suggesting that vicariously conditioned responses are goal-directed and not a mere mimicry of an observed action (Heyes, 1994; Heyes & Saggerson, 2002). However, despite these data, evidence for the effectiveness of observational learning is inconsistent. Church (1959) found that rats observing lights predicting a shock to a model do not generalize these contingencies to their own risk preferences.

Several critical differences can be highlighted between vicarious and active value learning, which may lead to differences in information acquisition. One factor is motivation, of key importance in Bandura’s (1977) social learning theory, given that passive observers do not directly incur costs or benefits during learning. Our emotional responses, enhanced when we act and experience outcomes ourselves, motivate our learning and decision-making (e.g. Schwarz & Clore, 1983). Anticipated emotions may also increase attention and an incentive to learn, and are likely to be greatest when actively learning. Alternatively, our emotions can potentially distract from, or “crowd out”, our goals (Loewenstein, 1996), or bias our memory for the frequency of past events (cf. emotional biases of eyewitness testimonies, e.g. Loftus, 1996), both of which could disrupt learning. Consistent with this “dark side of emotion”, individuals with decreased emotional responses for outcomes of risky decisions can show more advantageous decision-making (Shiv, Loewenstein, & Bechara, 2005).

Operant and observational learning differ in how attention is directed during learning. An actor’s ability to selectively sample an environment facilitates learning of an existing ‘region of uncertainty’ (Cohn, Atlas, & Ladner, 1994). Observers, on the other hand, lack this sampling control, making learning potentially inefficient. Observational learning may require a more explicit, declarative acquisition of knowledge, which may not be necessary given the procedural nature of operant learning (Howard, Mutter, & Howard, 1992; Kelly, Burton, Riedel, & Lynch, 2003; Willlingham, 1999). Neumann (1990) has argued that perception needs to be concurrently tied with action to influence subsequent behavior. fMRI studies, and assessments of learning deficits in Parkinson’s patients, support a functional dissociation of declarative or observational learning from non-declarative, procedural learning (Ostlund & Balleine, 2007; Poldrack et al., 2001; Shohamy et al., 2004). Furthermore, while explicit knowledge acquisition may be subject to distraction by other motivations, implicit learning of action-outcome associations may be less vulnerable to distraction (Neumann, 1990). From these considerations it is reasonable to predict superior learning through action than through observation.

In this study, our aim was to make a controlled comparison between active and observational learning in the context of human probabilistic value learning. Thus, we implemented a learning task where individuals learnt either by active sampling (with associated reward and punishment) or by passive observation. We assessed learning efficacy as shown by goal-directed choices and individuals’ explicit estimates of value. All aspects of the tasks, save for the critical factor of self versus other choice, were matched across two modes of value learning. Specifically, differences in attention and information were controlled, as participants could track the same sequences of outcomes in both learning conditions, as was motivation to learn, since participants earned money according to learning performance in both conditions.

## 2 Experiment 1

### 2.1 Participants

In this first experiment we recruited 17 healthy participants, screened for neurological or psychological disorders. Participants failing to reach a criterion of 60% accuracy by the end of each session, when choosing between the 80/20 probability of winning pair, were excluded from further analysis, given a performance level barely exceeding chance (i.e. 50% accuracy) and was considered as a failure to engage sufficiently with the task. This was the case only for one participant, leaving 16 participants for the full analysis (nine female, mean age 23.8 yrs, SD 3.0). Participants provided informed consent, according to UCL Research Ethics Committee approved procedures.

### 2.2 Procedure

Participants completed two sessions on consecutive days. In the first (the ‘actor session’), participants made choices between four stimuli (letters from Agathodaimon font), presented in different pairs on each trial, while concurrently attempting to learn the probability of winning from each. Participants were made aware that each stimulus was associated with a discrete and constant probability of winning (p{win}), and outcomes of each stimulus were drawn independently on every trial. Outcomes of chosen and unchosen stimuli were then shown sequentially, with yellow and red boxes indicating winning and losing outcomes, respectively. Critically, these outcomes directly influenced participant’s earnings for the actor session (with £1 awarded for each chosen win from 10 randomly selected trials). Participants were instructed to choose the stimulus with the highest p{win} on every trial in order to maximize earnings.

On day 2 (the ‘observer session’), participants learned the values of a novel set of stimuli (stimulus sets were balanced between sessions and across participants). We gave an instruction that this time participants would observe choices made previously by another participant, along with their associated outcomes. Participants were not provided with any information about this other participant, but were informed that these were real choices made by a different individual in a prior session. Participants were informed that, although they could learn from the outcomes of observed choices, these outcomes would *not* influence their own earnings for the observer session. Unknown to them, participants observed the sequence of choices they had made in their previous actor session, although now with visually novel stimuli. The two sessions were, therefore, matched in terms of the information from which they learned. Observer sessions were completed on day 2 in order to reduce memory for previous choice sequence. To match for motor responses, observers indicated the observed choice on each trial with a button-press. Since learning could not be measured in these observation trials, because a free choice is not made, we introduced test trials to assess learning in both actors and observers. These comprised nine blocks of trials (test blocks) at regular intervals throughout learning. Here, free choices were made by both actors and observers in the absence of outcome feedback (to prevent further learning).

**Fig. 1:**
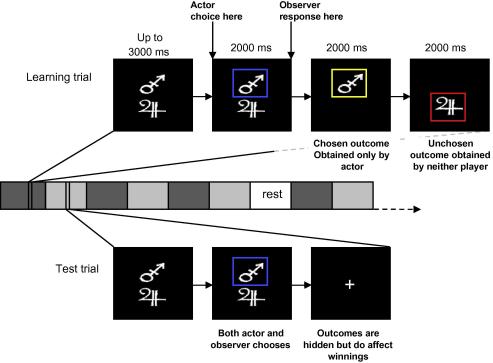
Timeline for both actor session and observer session. Learning blocks (dark gray) and test blocks (light gray) alternate nine times, with rests at three regular intervals. In learning trials, actors make a free choice between a stimulus pair, indicated by the blue box. Outcomes of the chosen and unchosen stimulus are then displayed sequentially, with a yellow box indicating a win, and red indicating no win. In observer sessions, learning trials differ only in participants’ response. Here, participants wait until the blue box is shown, indicating the “other participant’s” choice, and then press the button corresponding to the selected stimulus. Outcomes are presented as in the actor session. In test trials, free choices between stimulus pairs are made by both actors and observers, but outcomes are not displayed.

Fig. 1 illustrates exemplar learning and test trials and indicates the sole difference between actors and observers at the time of choice. Participants played a total of 324 trials per session (i.e. actor or observer). There were 12 trials in each of nine learning blocks, allowing for six presentations of each stimulus per block. In learning blocks, each stimulus could be presented within any possible pairing (i.e. six possible pairings, each pair presented nine times, resulting in 54 presentations overall for each of the four stimuli). There were 24 trials in each of the nine test blocks, allowing for 12 presentations of each stimulus. Stimulus pairings in test blocks were restricted to those of 80/20, 80/60, 60/40 and 40/20 proportions, which allowed for three repetitions of each pair type per test block. While 80/20 stimuli have a large discrepancy in probability, 80/60, 60/40 and 40/20 are matched. By using two levels of probability discrepancy (i.e. not including 80/40 and 60/20 gamble pairs in test trials), we maximize power for distinguishing an effect of discrepancy while preserving power to examine learning effects for each choice pair. Stimulus pairs were presented in a random order. Trial sequence was identical across actor and observer sessions and all pairings had equal frequency. At the end of each session, participants provided explicit estimates of p{win} for each stimulus learnt through either action or observation. Here, participants were shown each stimulus in turn and asked to explicitly write down their estimate of the probability of winning (as a percentage of trials) for the stimulus independent of its pairing.

In the observer session, participants were paid based on the (hidden) outcomes of 10 choices from the test trials. In their actor session, earnings were based on the chosen outcomes of five test and five learning trials. This matched the overall financial incentives across each learning session overall. Full payment was given after the second session, but participants were informed that the earnings of each session were independent. Practices for both actor and observer sessions were given at the beginning of the first session.

### 2.3 Design and analysis

We measured choice accuracy for each pair, over the nine test blocks, as the proportion of times that that option with the highest p{win} of each pair was chosen. Analysis was restricted to test blocks where both actors and observers made measurable free choices. We used a 2 × 4 × 9 within-subject design with factors for learning session (A/O), gamble pair (80/20, 80/60, 60/40, 40/20) and test block (1–9). To eliminate differences in individual learning ability, we measured within-subject changes in choice accuracy between the two sessions. Analyses were two-tailed to test for both increases and decreases in learning against the null hypothesis of no significant change between the two learning sessions.

Reaction times (RTs) were analyzed using a 2 × 2 × 9 ANOVA with factors comprising learning session (A/O), size of probability discrepancy (80/20 versus 80/60, 60/40 and 40/20) and test block (1–9). We predicted an effect of probability discrepancy on RT, since 80/20 pairs were considered to allow for easier value discrimination than 80/60, 60/40 and 40/20 pairs. We also tested for an effect of session on explicit estimates of p{win} for each stimulus, using a 2 × 4 ANOVA with factors for learning session (A/O) and stimulus (80, 60, 40, 20).

### 2.4 Results

**Fig. 2:**
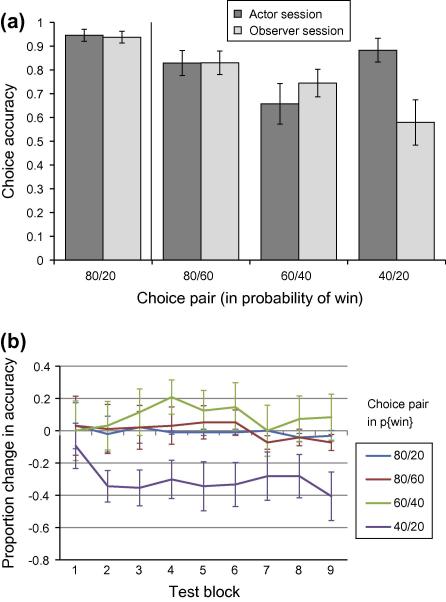
In (a) choice accuracy for each test trial gamble pair is shown collapsed across test block. In (b) the change in choice accuracy from actor to observer learning sessions (observer accuracy–actor accuracy) is plotted separately for each of the nine test blocks. Pairs are labeled according to the probability of a win for each stimulus. Choice accuracy is measured as the probability that participants chose the stimulus with the highest probability of a win. Actor and observer learning differed only for the 40/20 p{win} pair, with observers showing significantly lower accuracy compared to actors. Error bars show the standard error of the mean.

A repeated-measures ANOVA showed a main effect of the gamble pair on accuracy (*F*[3, 45] = 7.41, *p < *0.001, *η^2^ *= 0.33), an effect that also interacted significantly with session (*F*[3, 45] = 3.76, *p < *0.02, *η^2^ *= 0.20). Post-hoc paired *t*-tests showed this interaction was driven by a difference in actor and observer accuracy for the 40/20 pair alone, such that observers were significantly less accurate for these decisions (*t*[15] = 3.0, *p < *0.01) (Fig. 2a).We also found a quadratic effect of gamble pair in the case of actors (*F*[1, 15] = 13.05, *p *< 0.005, *η^2^ *= 0.47), which was not present for observers (gamble pair × session, *F*[1, 15] = 5.86, *p *< 0.05, *η^2^ = *0.28). This may reflect decreased uncertainty, and therefore higher accuracy, when choices involve the highest and lowest probabilities, similar to a payoff variability effect (see review by Erev and Barron (2005)). In contrast, actors perform relatively poorly in the case of the 60/40 pair, where outcome variance is highest.

We found a significant linear effect of learning over the nine test blocks (*F*[1, 15] = 15.09, *p < *0.002, *η^2^ = *0.50), such that accuracy improved over time. This effect interacted significantly with gamble pair (*F*[1, 15] = 9.05, *p < *0.01, *η^2^ *= 0.38), with accuracy improving more steeply for 80/20 and 80/60 pair choice, than for the two remaining pairs. There was no interaction of session × gamble pair × test block, suggesting that observers’ low choice accuracy for the 40/20 pair was not modulated by time (See Fig. 2b). The overall frequencies of choosing each stimulus over time are presented in Fig. S1.

Since the 60% and 40% win options were presented to participants both in the context of a better and a worse alternative option, we additionally examined the effect of this contextual pairing with a 2 × 2 × 2 within-subjects ANOVA with factors for session (A/O), choice (60/40) and context (whether the choice is the higher or lower value). Actors chose 60% and 40% options more frequently overall (*F*[1, 15] = 7.87, *p *< 0.02, *η^2^ *= 0.34). Generally, 60% and 40% options were selected significantly more when they were the highest value option in the pair (*F*[1, 15] = 105.75, *p *< 0.001, *η^2^ *= 0.88). Observers were significantly less likely to choose the 40% options when presented in a 40/20 pairing (mean 40% under 40/20 actor = 0.88; mean 40% under 40/20 observer = 0.58; *t*[15] = 2.97, *p < *0.01). This effect was not significant for the 60% option when presented in a 60/40 pairing (i.e. when 60% was the highest value stimulus) – (mean 60% under 60/40 actor = 0.66; mean 60% under 60/40 observer = 0.74; *t*[15] = −0.82, ns), nor were there any significant choice frequency difference between actor and observer sessions when 60% or 40% were the lower value stimulus in the pair (mean 60% under 80/60 actor = 0.17; mean 60% under 80/60 observer = 0.17; mean 40% under 60/40 actor = 0.34; mean 40% under 60/40 observer = 0.26). This was reflected in a session × choice × context interaction (*F*[1, 15] = 7.87, *p *< 0.02, *η^2^ *= 0.34). These findings are therefore in keeping with an over-valuation specific to the worst 20% win option rather than evidence for a more generic contextual effect.

**Fig. 3:**
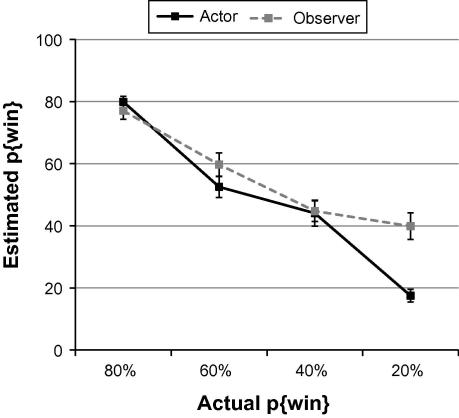
Participants’ estimated probability of a win (p{win}) for each stimulus, learned during the actor and observer sessions, plotted against the actual p{win} for each stimulus. Observers significantly overestimated the p{win} for the 20% win stimulus, compared to actors. Error bars show the standard error of the mean.

Participants’ explicit estimates of stimulus p{win} showed a specific impairment in learning in relation to lower p{win} options (Fig. 3). A repeated-measures ANOVA showed a gamble × session interaction in estimates of p{win} (*F*[3, 45] = 7.29, *p *< 0.0005, *η^2^ *= 0.33), such that p{win} for the 20% win option was significantly overestimated through observation compared to action (*t*(15) = 4.61, *p < *0.005). Observers’ individual choice preference in 40/20 test choices was also strongly associated with the degree to which the 20% win gamble was overvalued when observing compared to acting (*R*^2^ = 0.29, *p < *0.05).

Test trial RT’s were influenced by how much a gamble pair deviated in p{win}, such that participants were slower to choose between gambles with a close p{win} (80/60, 60/40, 40/20, mean = 1145.65 ms, SD = 54.37 ms) compared to an easily discriminable p{win} pair (80/20, mean = 959.67 ms, SD = 42.51 ms) (*F*[1, 15] = 125.81, *p < *0.0001, *η^2^ *= 0.89). There was also a linear effect of test number with participants becoming quicker with time (*F*[1, 15] = 35.65, *p < *0.0001, *η^2^ *= 0.70). There were no effects of session (mean actor = 1038.63 ms, SD actor = 51.01 ms; mean observer = 1066.69, SD observer = 49.67 ms), showing that any difference found between observational and operant learning was not explicable by RT differences.

### 2.5 Discussion

The results from Experiment 1 show that, while value learning through trial-and-error is highly accurate, observational learning is associated with erroneous learning of low-value options (i.e. those with the lowest probability of reward). In essence, observational learners show a striking over-estimation of the likelihood of winning from the lower-value options, a fallacy leading to impaired accuracy when choosing between two low-value options. This learning difference was apparent even though monetary incentives and visual information were matched in actor and observer learning. A different number of test trials were paid for observers relative to actors and this might have had a general effect on performance. However, it cannot explain observers’ asymmetrically poor accuracy when choosing between the 40/20 gamble pairs, and financial incentives were matched across each learning session overall. It is important to note that over-estimation of the value of the 20% win option did not cause observers to perform significantly worse when choosing between the 80/20 pairs. This is likely to reflect the fact that the probability difference is uniquely high for such pairs, allowing for lower uncertainty when determining the higher value choice.

It is interesting to observe that individual choice accuracies do not asymptote to 100%, as might be expected from rational decision makers once they accurately learn the value of stimuli. This may partially reflect the phenomenon of probability matching, a common finding in learning experiments (Herrnstein, 1961; Lau & Glimcher, 2005; Sugrue, Corrado, & Newsome, 2004), arising from a matching of choice frequency to average reinforcement rate. Note that, in our data, choice frequencies do not simply match learnt probabilities of reward, moreover probability matching does not in itself predict any difference between acting and observational learning.

Two potential design weaknesses can be identified in Experiment 1. First, by yoking the sequence of actor choices to participants’ subsequent observer session, to match actor and observer learning for information presented, we are not able to counterbalance session order. Since participants also learnt about novel stimuli in the second session, learning may be worse solely because the task has switched. To explicitly address these issues, we designed a second study (Experiment 2) to test for changes in learning between two actor sessions, with stimuli for each session taken from the equivalent sessions of Experiment 1. We predicted participants would show improved learning in the second actor session, despite the novel stimuli, due to generalization of learning strategy.

Secondly, in Experiment 1 it is impossible to distinguish between over-valuation of low-value options versus over-estimation of low probabilities. To address this, we conducted an additional experiment (Experiment 3) which reversed the framing of learning such that participants now learn in order to avoid losing, rather than to reap a reward. In so doing, options with the highest value were now associated with the lowest probability of losing, allowing us to explicitly dissociate probability and value.

## 3 Experiment 2

### 3.1 Participants

17 new participants took part in Experiment 2. As in Experiment 1, one participant was excluded due to a failure to reach our accuracy criterion. 16 participants remained (six female, mean age 31.2 yrs, SD 10.6).

### 3.2 Procedure and analysis

Here participants performed two actor sessions on consecutive days, using the same procedure and stimuli as in Experiment 1. As in Experiment 1, novel stimuli were used in the second session. Choice accuracy was measured as the probability that participants chose the stimulus with the highest probability of a win. Explicit estimates of p{win} were also assessed after each session.

While Experiment 2 used the same design as Experiment 1, critical analyses now involved the between-subject interactions in relation findings from Experiment 1. We term Experiment 1’s participants the AO group, and Experiment 2’s participants the AA group.

### 3.3 Results and Discussion

Within the AA group, we found a main effect of gamble pair (*F*[3, 45] = 5.64, *p < *0.005, *η^2^ *= 0.27), of test block (*F*[8, 120] = 4.36, *p < *0.001, *η^2^ *= 0.23), and a significant interaction of the two (*F*[24, 360] = 1.591, *p < *0.05, *η^2^ =* 0.10). While a significant main effect of gamble pair on accuracy was still apparent, this effect no longer interacted with session, suggesting that a poor performance in observational learning of low-value options cannot be explained by a session order effect. There was, however, a main effect of session (*F*[1, 15] = 6.40, *p < *0.05, *η^2^ *= 0.30), such that AA participants showed an improved accuracy from the first to the second session (see Fig. S2). Including a between-subject analysis against the AO participants of Experiment 1, we found a session × group interaction (*F*[1, 30] = 7.28, *p < *0.02, *η^2^ *= 0.20), and a session × gamble pair × group interaction (*F*[3, 90] = 3.68, *p < *0.02, *η^2^ *= 0.11), highlighting the specific impairment in observational learning for low-value options shown in Experiment 1.

Explicit estimates of p{win} were also more accurate in both sessions of the AA group. In the AA group, there was a significant main effect of gamble (*F*[3, 45] = 67.87, *p < *0.0001, *η^2^ *= 0.82) but the gamble × session interaction seen in Experiment 1 was no longer evident (see Fig. S3). When comparing experiments AO and AA, there was a significant session × group interaction (*F*[1, 30] = 7.59, *p < *0.01, *η^2^ *= 0.20) and a trend session × gamble × group interaction (*F*[3, 90] = 2.70, *p = *0.051, *η^2^ = *0.08), showing that the impaired estimation of the low-value options was specific to the observational learning session.

The results of Experiment 2 suggest that impaired learning in the observer session of Experiment 1 cannot be attributed to a temporal order effect or to the learning of novel stimuli. The AA group actually showed improved learning in the second session, perhaps attributable to generalization of learning strategies, but note this effect did not interact with gamble pair. This does not preclude the possibility, however, that a general improvement with task repetition may interact with the specific impairment we find in observational learning of low-value options. The significance of such an interaction cannot be determined in Experiment 1, however, since counterbalancing session order would have introduced the serious confound that sequences of choices would not have been matched between actor and observer learning.

## 4 Experiment 3

### 4.1 Participants

Sixteen new participants took part in Experiment 3 (seven female, mean age 21.1 yrs, SD 1.8).

### 4.2 Procedure and analysis

Experiment 3 was designed to distinguish between over-valuation of low-value options versus over-estimation of low probability events. By reversing the frame we change the valence and value of the corresponding outcome, while holding outcome probability constant. Hence, a 20% probability of a £1 win becomes a 20% probability of a £1 loss. Subjects overestimate the probability of the 20% win in Experiment 1, hence if they underestimate the probability of an 80% loss (i.e. the worst-valued option in both circumstances), this indicates a value-specific effect as distinct from an effect on probability (where we would expect over-estimation of the likelihood of both 20% win and loss outcomes). This manipulation in effect presents matched reward distributions, but translates the average reward for each from gain to loss.

Experiment 3 utilized the same procedure and tasks (both actor and observer) as those in Experiment 1, but with modified instructions and incentives. Participants were initially endowed with £10 per session. Instead of earning money from yellow boxes in the task, participants were informed that they would lose money from red boxes. In this way, the punishing power of the red boxes was assumed to attract more attention than in Experiment 1. At the end of the task, participants provided explicit estimates of the probability of losing (p{loss}) for each stimulus, in place of the p{win} estimates in Experiment 1.

Again, while Experiment 3 used the same design as Experiment 1, between-subject interactions with the findings from Experiment 1 were critical. We term Experiment 3’s participants the AO-loss group.

### 4.3 Results and discussion

Within the AO-loss group, we found main effects of session (*F*[1, 15] = 13.36, *p < *0.005, *η^2^ *= 0.47), gamble pair (*F*[3, 45] = 13.98, *p < *0.001, *η^2^ *= 0.48) and test block (*F*[8, 120] = 3.831, *p < *0.001, *η^2^ *= 0.20), as in the AO group. We also found an interaction of session × gamble pair (*F*[3, 45] = 12.15, *p < *0.0001, *η^2^ *= 0.45) which was, as in Experiment 1, driven by observers lower accuracy for the 40/20 p{win} pair compared to actors (*t*[15] = 5.89, *p < *0.0001) (see Fig. S4). The between-subject effect of group, i.e. Experiment 1 versus Experiment 3, interacted only with the main effects of session (*F*[1, 30] = 4.39, *p < *0.05, *η^2^ *= 0.13) and of gamble pair (*F*[3, 90] = 3.36, *p < *0.05, *η^2^ = *0.10). Therefore, the session × gamble pair interaction in choice accuracy, seen in Experiment 1, was replicated but now within the loss domain, with this effect being driven solely by observers’ impaired accuracy for the lowest value 40/20 win pair (now 60/80 loss pair).

In the explicit estimates, there was a significant main effect of session (*F*[1, 15] = 12.86, *p < *0.005, *η^2^ *= 0.46) and of gamble (*F*[3, 45] = 75.85, *p < *0.0001, *η^2^ *= 0.84), along with a gamble × session interaction (*F*[3, 45] = 8.87, *p < *0.0005, *η^2^ *= 0.37). Therefore, participants’ explicit estimates of p{loss} for each stimulus also replicated the results of Experiment 1, supporting an over-valuing of the lowest value options (i.e. participants underestimated p{loss} for the 80% loss option) rather than an over-estimation of small probabilities (participants showed high estimation accuracy for options with the lower p{loss}) (see Fig. S5). However, in the context of this argument, it is not obvious why the 40% win option was not also overvalued. One possibility is that the 20% win option may be qualitatively, as well as quantitatively, of lower value since it is the only option never paired with an option of an even lower value. This might explain why we find over-valuation only for the 20% win option, but we accept that this conjecture needs to be tested directly. In Experiment 3, we also found a slight undervaluation of 80% loss (*t*[15] = −2.48, *p *< 0.05). Observer accuracy when choosing between the 80/20 win pair also showed a trend to be lower than for actors (*t*[15] = 1.83, *p *< 0.1). The magnitude of this effect was much smaller than in the 20/40 condition and this asymmetrical effect cannot be explained solely by an error in probability assessment. However, this finding hints that both a large over-valuation for low-value options and also a smaller mis-estimation of low probabilities may be at play in Experiment 3.

## 5 General discussion

Experiments 1 and 3 both show an over-valuation for low-value options during observational learning, an effect evident across implicit (i.e. choice preference) and explicit indices of subjective value. This difference was evident despite the observational and operant learning tasks being matched for visual information, and for monetary incentives to learn. In contrast, Experiment 2 shows that learning is generally improved between two active learning sessions despite the time delay and the novel stimuli being learned. Experiment 3 also suggests that the deficit in observational learning is particularly in valuation of low-value options, rather than an imprecision when estimating low probabilities, indicating that observers are biased to (inappropriately) discount the chance they will experience the negative outcomes seen to be incurred by others. These results are intriguing since neither social learning theories nor reinforcement learning approaches explicitly predict that action-outcome contingency learning should depend upon the manner through which they are learnt. Also recent neuroimaging studies in humans report neuronal responses to errors (Koelewijn, van Schie, Bekkering, Oostenveld, & Jensen, 2008; van Schie, Mars, Coles, & Bekkering, 2004; Yu & Zhou, 2006) and successes (Mobbs et al., 2009) observed from the behavior of others, comparable to those seen in response to self-experienced outcomes, meaning one might predict little difference in learning from such responses. Yu and colleagues report feedback-related negativities (FRN) that are smaller in magnitude, more posteriorly located in the brain and have a smaller impact on future behavior in observation compared to action, consistent with the learning differences we find (Yu & Zhou, 2006). While they suggest that these differences may be related to decreased motivation and emotional involvement in the outcome during observation, to our knowledge our present data are the first to indicate that observational learning may be suboptimal in the context of low-value options.

The learning deficit shown by observers is equivalent to a behavioral manifestation of an optimistic bias, reflecting a tendency to underweight the prospect of a negative experience. Optimism often has a socially comparative nature as when we tend to overestimate our own strengths and resources, while discounting those of others (Radcliffe & Klein, 2002). This bias is likely to be associated with the protection of self-esteem and avoidance of social anxiety (e.g. Hirsch & Mathews, 2000), coupled with a desire to be better than others (Weinstein, 1989). Highly optimistic individuals are known to retain less information on personal risk factors and also show more initial avoidance of such information, while those with lower optimism were more realistic and more open to receiving risk information (Radcliffe & Klein, 2002). We show that observers overvalue options that they have seen resulting in losses for others, reflecting a similarly optimistic judgment of personal risk. It is important to note that, with our task design, we cannot determine whether the over-valuation of low-value options is of a socially comparative rather than of a non-social nature. This remains a critical point to address in future studies, using experimental designs aimed at teasing apart these two possible underlying influences.

In contrast to our findings, Braver and Rohrer (1978) found that observers learnt appropriate (i.e. reinforced) responses better than did actors (participants) in the context of interpersonal conflict resolution in a prisoner’s dilemma game. They proposed that the difference may be due to an actor’s reluctance to modify their behavior in response to their failures, instead attributing responsibility for the failure externally (Jones & Nisbett, 1971). However, it has also been suggested that egocentrism (i.e. internal focus of attention, and failure to carefully consider the circumstances of others) encourages a particular tendency to feel that one is less likely to experience the negative events experienced by others (Weinstein & Lachendro, 1982), known as comparative optimism. There is a recognized tendency for individuals to show an external attribution for failures and an internal attribution for successes, a bias that might interfere with accurate learning of action-outcome contingencies. Specifically, such an attribution bias distorts observational learning through a tendency to attribute an observed actor’s failures to internally (i.e. dispositional) causes, encouraging an observer to believe they are less likely to fail or lose themselves. On the other hand, the actor’s successes are perceived as externally determined, easily obtainable, and not due to any exceptional skill in the actor.

While these optimistic biases, whether social or non-social, can lead to a selective encoding of positive information, and underweighting of negative outcomes, learning through direct experiment can lead to increased realism in estimating risk (Burger & Palmer, 1992; Helweg-Larsen, 1999; Van der Velde, Van der Pligt, & Hooykaas, 1994; Weinstein, 1987, 1989). This may reflect the greater vividness and self-relevance of direct experience (Helweg-Larsen, 1999; Stapel & Velthuijsen, 1996) or reflect improved recall of one’s own actions (Weinstein, 1987, see also Tversky & Kahneman’s availability heuristic, 1974). Such an interpretation accords with findings that directly experienced information is given greater weight than observed information in guiding future behavior in social games, even if both are equally informative and equally attended (Simonsohn, Karlsson, Loewenstein, & Ariely, 2008).

An alternative explanation to account for the disparity between observational and operant learning might be that learning about low-value options is simply more difficult, a difficulty amplified by the relatively greater declarative demands of observational learning. However, the success rate for observer learning of the 20% win option did not increase at all over the nine test blocks, suggesting that learning was not simply slower in observers. Another possibility is that the effect could be explained by differences in sampling between operant and observational learning. While sampling errors have been implicated in biased probability weightings, such results show a tendency to overweight high probability gains when learning through experience (e.g. Hertwig, Barron, Weber, & Erev, 2004), in contrast to our finding of overweighting low probability gains. Moreover, a difficulty for a sampling difference explanation is the fact that full and identical feedback, of chosen and unchosen gamble outcomes, was presented to both actors and observers. However, sampling errors may occur at the level of attention rather than choice, where certain outcomes may be deemed to hold more personal relevance than others. The active nature of operant learning could also engage the actor and improve efficiency of learning (Cohn et al., 1994), although this would be predicted to occur across the *full* range of probabilities.

In this article, we demonstrate a difference in value learning between acting and observation, an effect not previously reported to the best of our knowledge. These findings have important implications for how we apply learning theory to vicarious learning, either social or non-social, as classical models assign no differences to these alternative models of learning. This bias in learning indicates that action-outcome contingency learning depends on the manner through which it is learned, and indicates that actors and observers implement different weightings for positive and negative experiences as they sample outcomes. As we are interested in the mechanisms underlying this effect, we excluded two important alternative explanations. In Experiment 2 we rule out a value-specific order effect on learning, while in Experiment 3 we show that this effect is driven by poor estimation of value rather than of probability. This leaves open a possibility that the effect reflects an optimistic bias in observational learning leading one to underestimate the likelihood of experiencing negative events, as observed occurring to others, a bias not present in actors learning by direct experience as in trial-and-error. To provide a more precise account we believe requires additional experimentation. In particular, the fact that the effect is specific to the lowest value option of the choice set (i.e. only the 20% win option) could indicate that this over-valuation is a non-linear effect of value learning present over-and-above a certain threshold. This non-linear effect may also be explained by a critical role of context in value learning, whereby observers’ over-valuation is only for options that are of low value relative to either the whole choice set (i.e. 20% win options were the lowest value in the choice set) or to the alternative option in the pair (i.e. 20% win options were the only option never paired with an option of an even lower value). Indeed such reference dependent effects on subjective representations of value are supported by an extensive psychological (e.g. Kahneman & Tversky, 1979; Mellers, 2000) and neuroscience literature (e.g. Breiter, Aharon, Kahneman, Dale, & Shizgal, 2001; Elliott, Agnew, & Deakin, 2008; Tremblay & Schultz, 1999).

## Appendix A Supplementary material

Supplementary data 1 Fig. S1. With data from Experiments 1 and 3 collapsed for this plot, the lines show the probability with which participants choose the 80%, 60%, 40% and 20% win options, in each of the nine test blocks, given that they are each presented with the same frequency. Choice probability is shown for actor learning (filled lines) and observer learning (broken lines). Unconnected dots show the actual cumulative frequency of wins for each stimulus in the learning trials preceding each test block, which are identical for actors (diamonds) and observers (crosses). Actors can be seen to learn quickly the low value of choosing the 20% win option, avoiding this choice on the majority of trials, while observers show a higher tendency to choose this option. Actors also demonstrate an apparent over-valuation of the 40% option, likely owing to their accurate realization that it is of a higher value than the 20% win option and resulting from an attempt to polarize the two values. Error bars show the standard error of the mean.

Supplementary data 2 In a) choice accuracy for test trial gamble pairs in experiment 2’s AA group is shown collapsed across test block. In b) the change in choice accuracy from the first to the second actor learning session (second actor accuracy–first actor accuracy) is plotted separately for each of the nine test blocks. Pairs are labeled according to the probability of a win for each stimulus. Error bars show the standard error of the mean.

Supplementary data 3 Fig. S3. Estimated probability of a win (p{win}) for each stimulus, learned during the first and second actor sessions of Experiment 2’s AA participants, plotted against the actual p{win} for each stimulus. Error bars show the standard error of the mean.

Supplementary data 4 Fig. S4. In a) choice accuracy for test trial gamble pairs in experiment 3’s AO-loss group is shown collapsed across test block. In b) the change in choice accuracy from actor to observer learning sessions (observer accuracy–actor accuracy) is plotted separately for each of the nine test blocks. Pairs are labeled according to the probability of a win for each stimulus. Error bars show the standard error of the mean.

Supplementary data 5 Fig. S5. Estimated probability of a win (p{loss}) for each stimulus, learned during the actor and observer sessions of Experiment 3’s AO-loss participants, plotted against the actual p{loss} for each stimulus. Error bars show the standard error of the mean.
