# Supplementary figures and images for: Optimistic biases in observational learning of value
Source: Cognition. 2011 Jun;119(3):394–402. doi: 10.1016/j.cognition.2011.02.004 (PMC3081069; doi:10.1016/j.cognition.2011.02.004)

## Slide 1
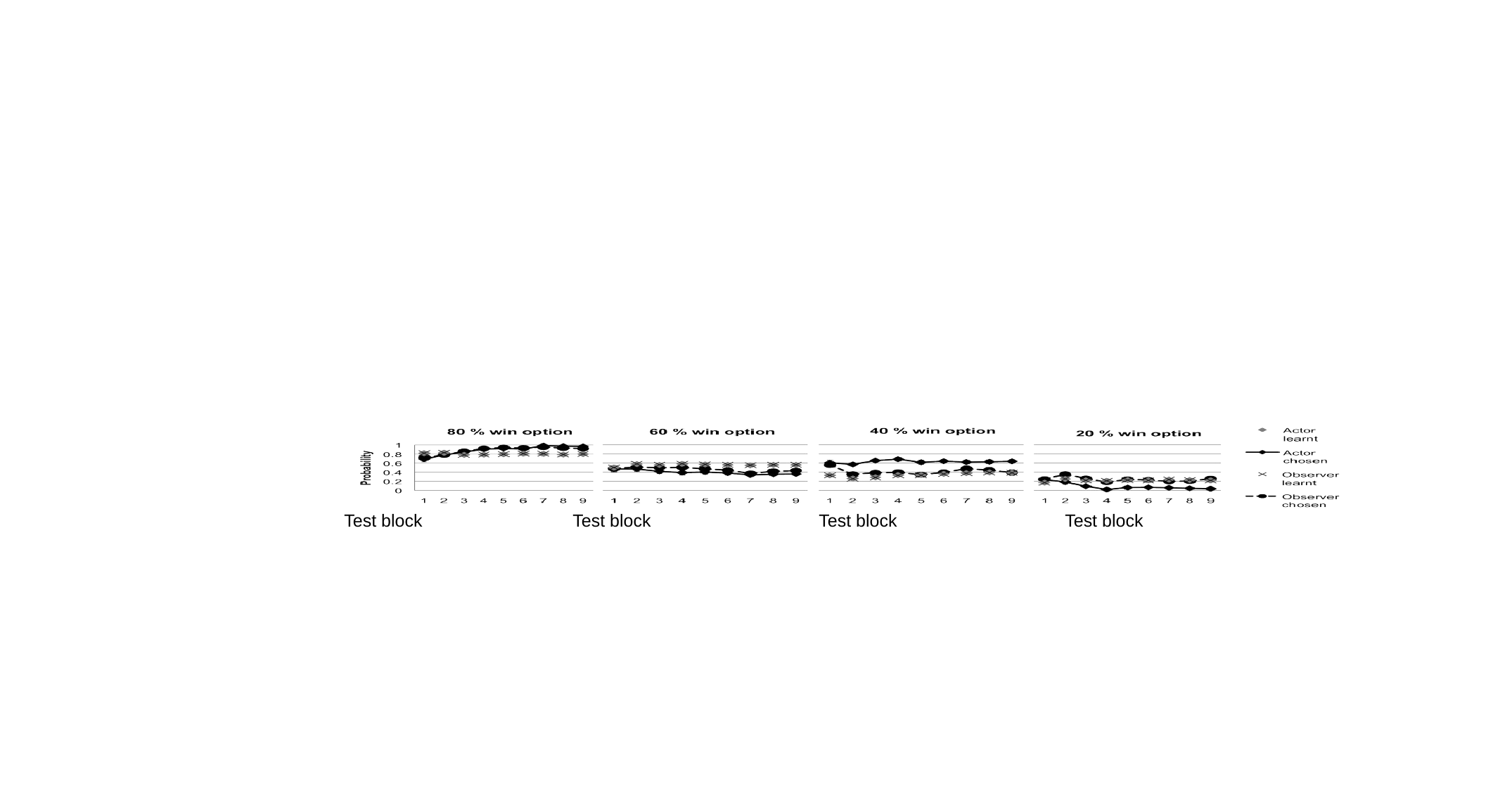

Test block
Test block
Test block
Test block

Supplement: Supplementary data 1 — Fig. S1. With data from Experiments 1 and 3 collapsed for this plot, the lines show the probability with which participants choose the 80%, 60%, 40% and 20% win options, in each of the nine test blocks, given that they are each presented with the same frequency. Choice probability is shown for actor learning (filled lines) and observer learning (broken lines). Unconnected dots show the actual cumulative frequency of wins for each stimulus in the learning trials preceding each test block, which are identical for actors (diamonds) and observers (crosses). Actors can be seen to learn quickly the low value of choosing the 20% win option, avoiding this choice on the majority of trials, while observers show a higher tendency to choose this option. Actors also demonstrate an apparent over-valuation of the 40% option, likely owing to their accurate realization that it is of a higher value than the 20% win option and resulting from an attempt to polarize the two values. Error bars show the standard error of the mean. [file mmc1.ppt]

## Slide 1
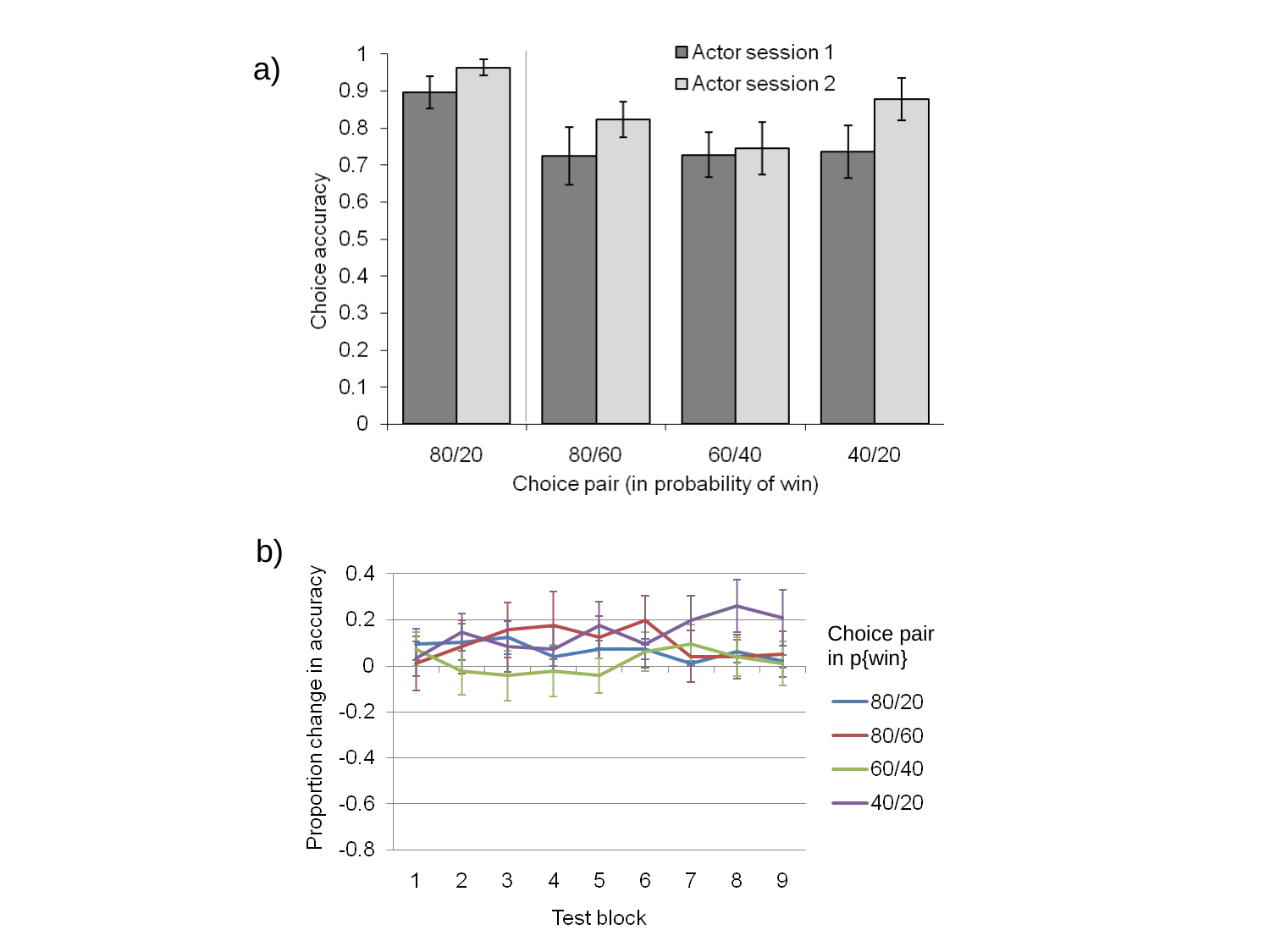

a)
b)
Choice pair in p{win}

Supplement: Supplementary data 2 — In a) choice accuracy for test trial gamble pairs in experiment 2’s AA group is shown collapsed across test block. In b) the change in choice accuracy from the first to the second actor learning session (second actor accuracy–first actor accuracy) is plotted separately for each of the nine test blocks. Pairs are labeled according to the probability of a win for each stimulus. Error bars show the standard error of the mean. [file mmc2.ppt]

## Slide 1
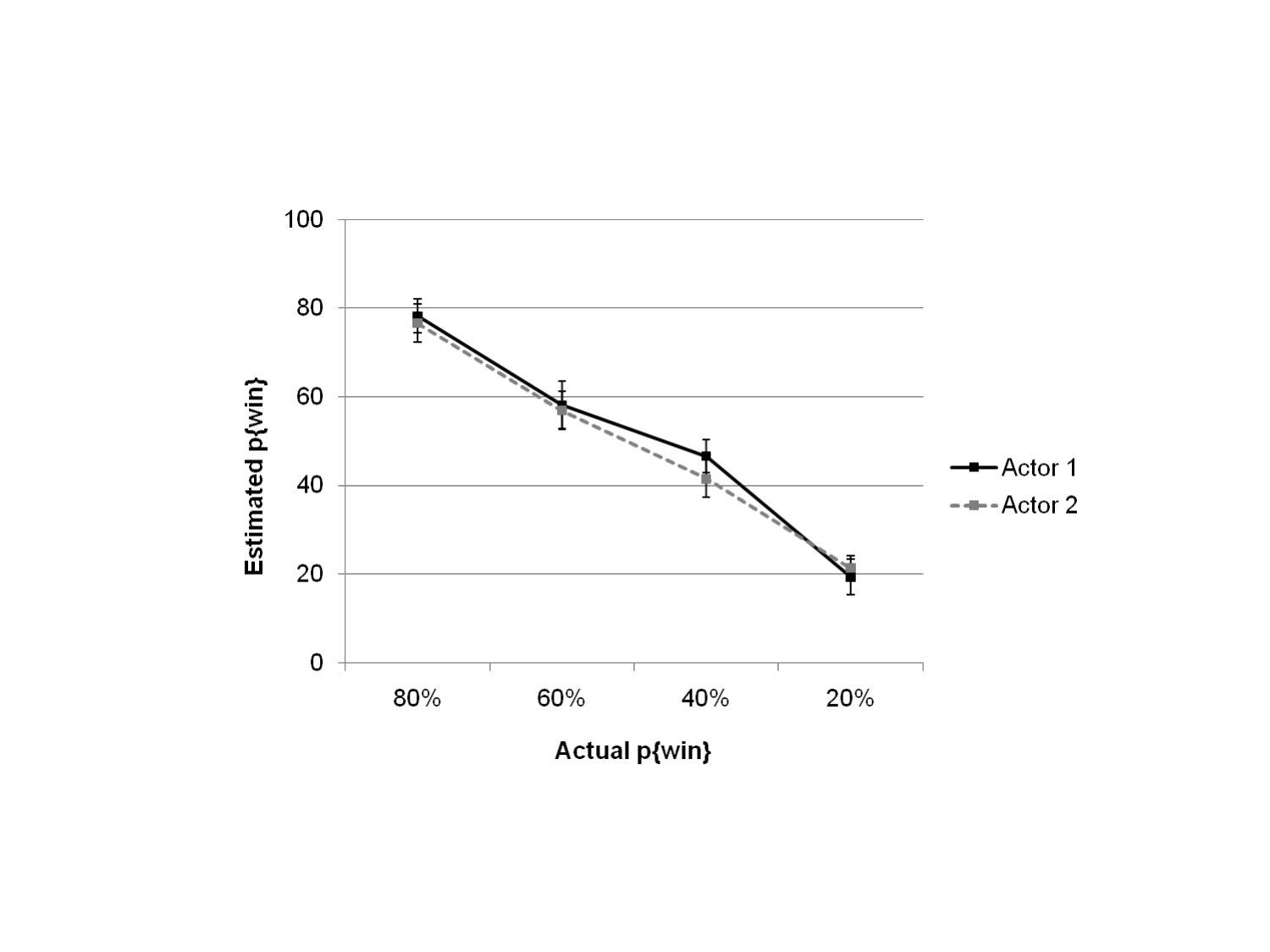

Supplement: Supplementary data 3 — Fig. S3. Estimated probability of a win (p{win}) for each stimulus, learned during the first and second actor sessions of Experiment 2’s AA participants, plotted against the actual p{win} for each stimulus. Error bars show the standard error of the mean. [file mmc3.ppt]

## Slide 1
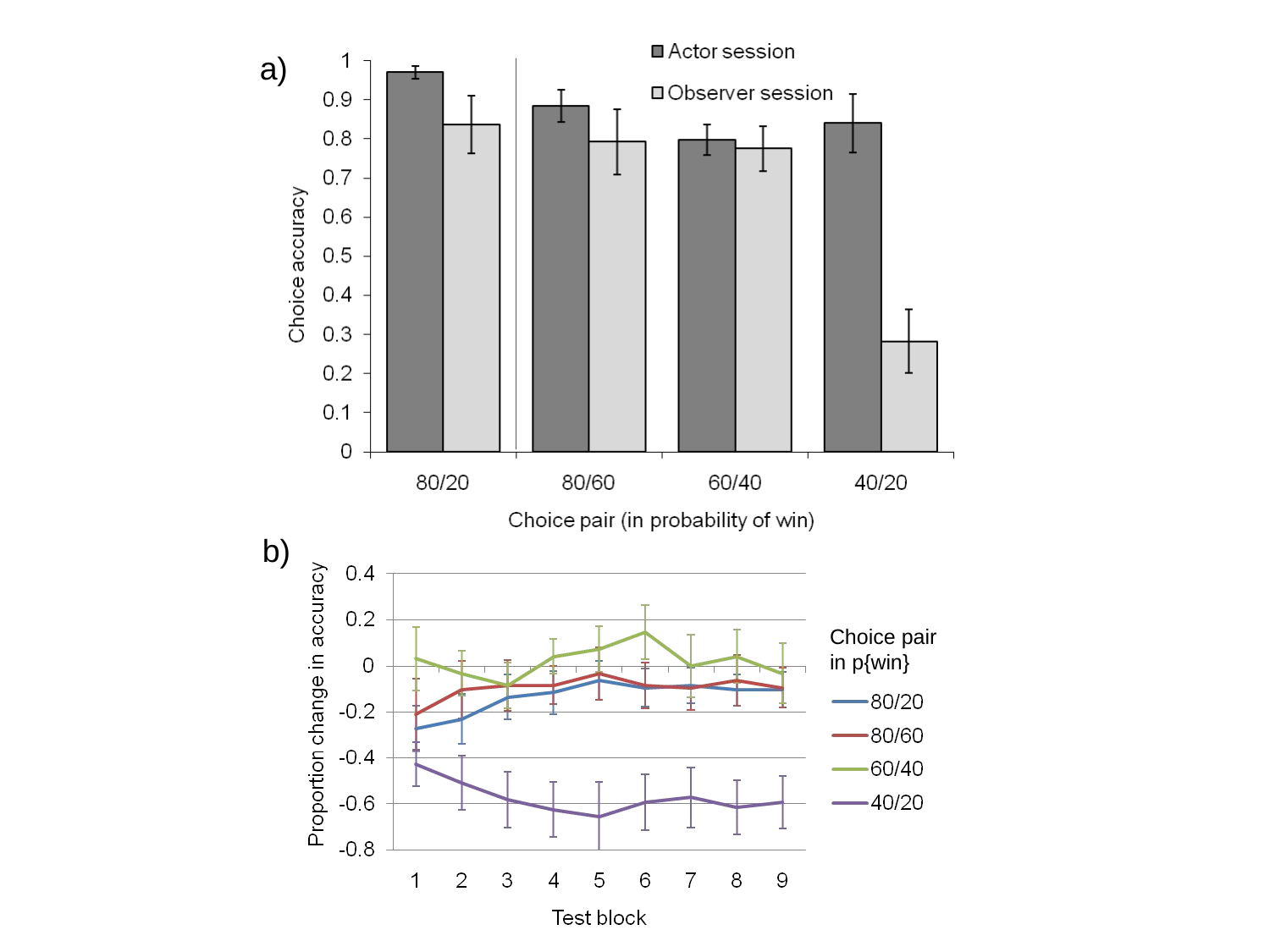

a)
b)
Choice pair in p{win}

Supplement: Supplementary data 4 — Fig. S4. In a) choice accuracy for test trial gamble pairs in experiment 3’s AO-loss group is shown collapsed across test block. In b) the change in choice accuracy from actor to observer learning sessions (observer accuracy–actor accuracy) is plotted separately for each of the nine test blocks. Pairs are labeled according to the probability of a win for each stimulus. Error bars show the standard error of the mean. [file mmc4.ppt]

## Slide 1
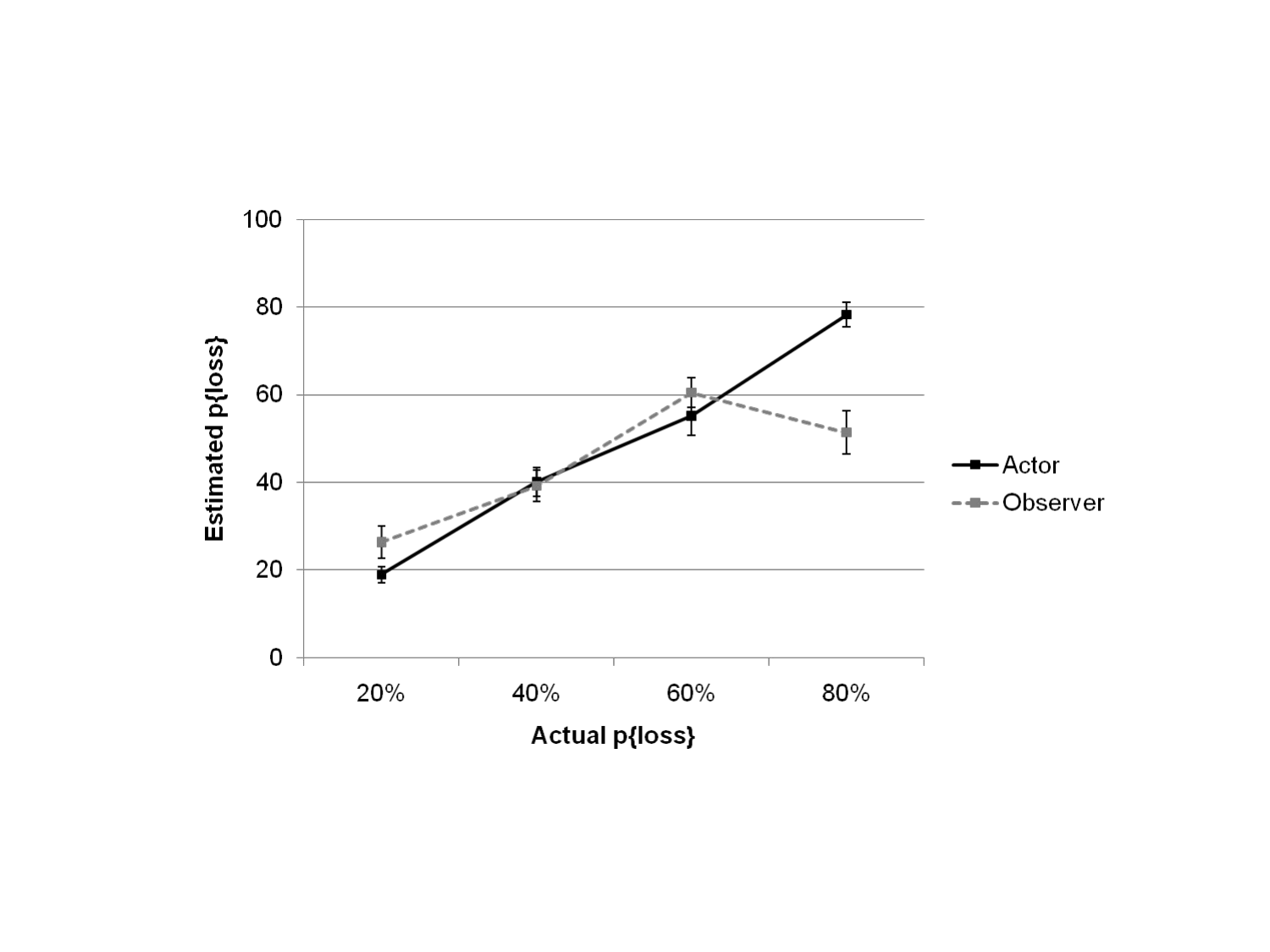

Supplement: Supplementary data 5 — Fig. S5. Estimated probability of a win (p{loss}) for each stimulus, learned during the actor and observer sessions of Experiment 3’s AO-loss participants, plotted against the actual p{loss} for each stimulus. Error bars show the standard error of the mean. [file mmc5.ppt]
